# Major Gene for Field Stem Rust Resistance Co-Locates with Resistance Gene *Sr12* in ‘Thatcher’ Wheat

**DOI:** 10.1371/journal.pone.0157029

**Published:** 2016-06-16

**Authors:** Colin W. Hiebert, James A. Kolmer, Curt A. McCartney, Jordan Briggs, Tom Fetch, Harbans Bariana, Frederic Choulet, Matthew N. Rouse, Wolfgang Spielmeyer

**Affiliations:** 1 Agriculture and Agri-Food Canada, Cereal Research Centre, Morden, Manitoba, Canada; 2 Department of Plant Pathology, University of Minnesota, Saint Paul, Minnesota, United States of America; 3 United States Department of Agriculture–Agriculture Research Service, Cereal Disease Laboratory, Saint Paul, Minnesota, United States of America; 4 Agriculture and Agri-Food Canada, Brandon Research Centre, Brandon, Manitoba, Canada; 5 Plant Breeding Institute, University of Sydney, Cobbitty, New South Wales, Australia; 6 INRA UMR1095 Genetics, Diversity and Ecophysiology of Cereals, Clermont-Ferrand, France; 7 Commonwealth Scientific and Industrial Research Organisation—Agriculture and Food, Canberra, Australian Capital Territory, Australia; University of California Davis, UNITED STATES

## Abstract

Stem rust, caused by *Puccinia graminis* (*Pgt*), is a damaging disease of wheat that can be controlled by utilizing effective stem rust resistance genes. ‘Thatcher’ wheat carries complex resistance to stem rust that is enhanced in the presence of the resistance gene *Lr34*. The purpose of this study was to examine APR in ‘Thatcher’ and look for genetic interactions with *Lr34*. A RIL population was tested for stem rust resistance in field nurseries in Canada, USA, and Kenya. BSA was used to find SNP markers associated with reduced stem rust severity. A major QTL was identified on chromosome 3BL near the centromere in all environments. Seedling testing showed that *Sr12* mapped to the same region as the QTL for APR. The SNP markers were physically mapped and the region carrying the resistance was searched for sequences with homology to members of the NB-LRR resistance gene family. SNP marker from one NB-LRR-like sequence, *NB-LRR3* co-segregated with *Sr12*. Two additional populations, including one that lacked *Lr34*, were tested in field nurseries. *NB-LRR3* mapped near the maximum LOD for reduction in stem rust severity in both populations. Lines from a population that segregated for *Sr12* and *Lr34* were tested for seedling *Pgt* biomass and infection type, as well as APR to field stem rust which showed an interaction between the genes. We concluded that *Sr12*, or a gene closely linked to *Sr12*, was responsible for ‘Thatcher’-derived APR in several environments and this resistance was enhanced in the presence of *Lr34*.

## Introduction

Stem rust of wheat, caused by the fungus *Puccinia graminis* Pers.:Pers. f. sp. *tritici* Eriks. & E. Henn. (*Pgt*), is capable of causing devastating grain yield losses. For example, during the last North American stem rust epidemic between 1953–55 up to 40% of the spring wheat crop was lost because of the virulent *Pgt* race 15B [1]. Resistant cultivars have effectively controlled stem rust for several decades. More recently, new races of *Pgt* were detected in Africa, specifically Ug99 and its variants that are broadly virulent on a majority of the wheat varieties grown globally [2–6]. This resulted in increased interest and investment in understanding the genetics of stem rust resistance in wheat.

The hard red spring wheat cultivar ‘Thatcher’ [7] was the dominant cultivar in the Canadian Prairies from 1939 to the mid-1960s mainly due to its resistance to stem rust and excellent end-use quality [8]. Stem rust resistance in ‘Thatcher’ has been the subject of several genetic studies. Four seedling stem rust resistance (*Sr*) genes, *Sr5*, *Sr9g*, *Sr12*, and *Sr16* have been reported in ‘Thatcher’ [9,10]. However, the seedling resistance characterized in ‘Thatcher’ does not explain the adult-plant resistance (APR) observed in the field to North American races of *Pgt* [11]. Furthermore, ‘Thatcher’ shows a moderately resistant to moderately susceptible response in Kenyan field tests in the presence of Ug99 and its variants while showing a susceptible infection type at the seedling stage to the same field races demonstrating some level of APR to Ug99 [12].

*Lr34* is an important disease resistance gene as it confers confers resistance to leaf rust, stem rust, stripe rust, powdery mildew and barley yellow dwarf virus [13–18]. It has also been characterized as a ‘non-suppressor’ of stem rust resistance genes in ‘Thatcher’ and its derivatives [13–19]. A locus on chromosome arm 7DL suppresses some seedling stem rust resistance in ‘Thatcher’-derived wheats that are expressed when the suppressor is removed by chromosome deficiencies or by mutagenesis [20, 21]. The presence of *Lr34* appears to negate the effect of the suppressor [19], though a direct link between the actions of these loci has not been established. Regardless, *Lr34* enhances field resistance to *Pgt* in the ‘Thatcher’ background and in the cultivar ‘Roblin’ and ‘Pasqua’, and has been shown to interact with other *Sr* genes [22–24]. In our preliminary experiments and in other studies, a ‘Thatcher’ near-isogenic line (NIL) carrying *Lr34* (RL6058 = Thatcher*6/PI58548; Tc-*Lr34*) showed enhanced stem rust resistance compared to ‘Thatcher’ in North America, Australia and Kenya [25].

Other studies have also examined the interactions between *Lr34* and *Sr* genes found in ‘Thatcher’. One study suggested that *Sr16* and *Sr12* may have contributed to resistance of adult plants in their ‘Thatcher’-derived population but focused on resistance associated with markers on chromosome arm 2BL [26]. In a follow-up study, a QTL was identified in ‘Thatcher’ on chromosome arm 2BL that was enhanced by *Lr34* [25]. The peak of the QTL on 2BL was near the map location of *Sr9* [27, 28]. In a similar study, a QTL was found on chromosome 3B which corresponded to the map position of *Sr12* as determined by testing their population at the seedling stage with a race of *Pgt* that was avirulent to *Sr12* [12]. The above studies used populations that segregated for *Lr34* and suggested that *Sr12* plays an important role in the APR of ‘Thatcher’ [12, 26]. *Sr12* was assigned to the short arm of chromosome 3B by telocentric mapping; however the authors state that it is possible that *Sr12* could be near the centromere on the long arm [29]. None of the QTL mapping studies above addressed which arm of chromosome 3B is involved in Thatcher’s APR.

In this study, our objectives were to map stem rust resistance that interacts with *Lr34* in ‘Thatcher’ at the adult plant stage and to develop DNA markers that facilitate further characterization of the resistance and are useful for marker-assisted breeding.

## Materials and Methods

### Populations

An F_6:7_ recombinant inbred line (RIL) population consisting of 94 lines was developed from the cross of RL90/RL6058, which will be referred to as the RL population hereafter. RL6058 is a ‘Thatcher’ near-isogenic line (NIL) that carries *Lr34* and has a high level of resistance to stem rust. RL90 is a line selected from a RIL population developed from the cross of RL6071 (‘Prelude’/8*‘Marquis’*2/3/‘Prelude’//‘Prelude’/8*‘Marquis’) and RL6058 [25]. RL90 carries *Lr34* but shows a moderately susceptible to susceptible reaction to stem rust. An F_4_ family derived from a single F_3_ plant from the RL population (RL90-B17) that was heterozygous for markers flanking resistance on chromosome 3B was grown to maturity. The seed from this population was treated as an “F_2_-equivalent” population (RL-F_2_ population) and 110 lines were used to improve the map resolution of markers mapped in the RL population.

A doubled haploid (DH) population consisting of 137 lines was generated from the cross ‘Chinese Spring’/RL6058 using the maize pollination method [30] and will be referred to as the CS population hereafter. ‘Chinese Spring’ carries *Lr34* [14], but it is susceptible to the *Pgt* races used in this experiment while RL6058 is resistant.

Another RIL population of 95 lines was developed from the cross of ‘Kenyon’/861SMN-2137 and will be referred to as the KN population hereafter. ‘Kenyon’ (Neepawa*5/Buck Manantial) is a Canadian hard red spring wheat cultivar registered in the 1985 [31] and is resistant to stem rust in field tests in Canada and Kenya. Line 861SMN-2137 is resistant to stem rust in field tests in Canada but is susceptible to stem rust in Kenya. Neither parent carries *Lr34*.

To study interactions with *Lr34*, RL90 (carries *Lr34* and is susceptible to stem rust) was crossed to RL6077 (‘Thatcher’*6/PI250413; a near-isogenic line of ‘Thatcher’ carrying *Lr67*) to generate F_2_ seed which was screened as half seeds for markers flanking the chromosome 3B resistance locus (*IWA6086* and *IWA4613*) and the gene-based marker *csLr34SNP* for *Lr34* [32]. Three to four F_2_ plants were identified for each genotypic category (null, *Lr34*, *Sr12* region, and *Lr34*+*Sr12* region). F_3_ families were scored for stem rust resistance at the seedling stage using Australian *Pgt* race 98–1,2,(3),(5),6 and screened in the field nurseries in St. Paul in 2014 and Morden in 2015 (see below).

A final RIL population of 160 lines from the cross ‘McNeal’/‘Thatcher’ was used in this study. The population was previously reported [28] and will be referred to as the MN population hereafter. ‘McNeal’ (RS 6880/Glenman) is susceptible to the *Pgt* races used in this study and carries *Lr34*. This population was used previously to map *Sr12*.

In summary, the RL, CS and KN populations were used to map major resistance to *Pgt* in the field. The RL-F_2_ population was generated to improve map resolution for the chromosome 3B region and the RL90/RL6071 population was used to study interactions with *Lr34*. Markers developed in this study were also tested in the MN population to confirm tight linkage to the *Sr12* gene.

### Testing with *Pgt*

The RL, CS, and KN populations and F_3_ families from the RL90/RL6077 population were tested for stem rust resistance in replicated and randomized field trials. The RL population was tested in field nurseries in Morden, MB, Canada in 2014 and 2015 (80 RILs), Njoro, Kenya in 2013 (89 RILs), and St. Paul, MN, USA in 2013 (87 RILs). The CS population (137 RILs) was tested in the Morden nursery in 2014. The KN population of 95 lines was tested in Njoro, Kenya in 2013 and 2014. No specific permissions were required to conduct field trials in Canada, USA and Kenya.

In Morden, entries were planted in 1 m rows with ranges of five rows flanked by susceptible spreader rows. The spreader rows were inoculated with a mixture of *Pgt* races (TPMKR, TMRTK, RKQSR, RHTSJ, QTHJT, RTHJT and MCCFR with AAFC-MRDC isolate numbers 1373, 1311, 1312, 1562, 1347, 1561, and 1541 respectively; from the AAFC-MRDC *Pgt* collection of isolates found in Canadian fields) at the jointing stage by spraying urediniospores suspended in a light mineral oil on a day preceding anticipated overnight dew. Spores from heavily infected spreader rows caused infection of the experimental plots. The populations were rated for stem rust severity and infection response once the susceptible checks showed heavy disease (severities near 80%) which was approximately at anthesis. The modified Cobb scale was used to assess the populations [33]. In St. Paul a similar procedure was used except 2 m rows were planted, spreader rows were planted perpendicular to the experimental plots, and the race mixture included *Pgt* races QFCSC, QTHJC, MCCFC, RCRSC, RKQQC, and TPMKC (isolates 06ND76C, 75ND717C, 59KS19, 77ND82A, 99KS76A, and 74MN1409, respectively; from the USDA-CDL *Pgt* collection of isolates found in US fields). Of the above races, only MCCFR (in Canada) and QFCSC and MCCFC (in the USA) were avirulent to *Sr12*.

In Kenya, *Pgt* inoculum for the field was increased on lines carrying *Sr31* to select Ug99-type races. Experimental lines were planted in two 1 m rows and hills of plants containing a mixture of cultivars susceptible to Ug99 were grown next to the experimental plots. Spreader hills were inoculated at jointing. Plots were rated for stem rust severity at anthesis.

The RL, RL-F_2_ and MN populations were tested with *Pgt* at the seedling stage using races that are avirulent to *Sr12*. The RL population was inoculated with *Pgt* race MCCFR (isolate 1541, as above) by spraying a mixture of urediniospores in light mineral oil onto the seedlings after the first leaf was fully emerged. Seedlings were incubated in dew chambers for 16 hr and then treated with light for 3 hr in the dew chambers. Seedlings were grown in a greenhouse at approximately 20°C with 16 hr of light daily. Two weeks after inoculation seedlings were rated for their infection types (IT) following the commonly used ‘0’ to ‘4’ IT scale [34]. The MN population was inoculated with race SCCSC (isolate 09ID73-2) as previously described [12]. The same IT scale was used to assess the MN population. The RL and RL-F_2_ populations were inoculated with *Pgt* race 98–1,2,(3),(5),6 (an isolate from the University of Sydney, Plant Breeding Institute collection) by applying urediniospores mixed in talc onto seedlings and incubating in dew chamber for 24 hrs at approximately 18–20°C before transferring them to greenhouse set at approximately 18–20°C. Families were rated for their infection types 14 days post-inoculation (DPI) and were classified resistant, segregating, or susceptible.

The selected genotypically homozygous RL90/RL6077 F_3_ families were inoculated at the seedling stage using *Pgt* race 98–1,2,(3),(5),6 by mixing urediniospores and talc as described above. Ten days after inoculation tissue from the first leaf was harvested and chitin content was measured to determine fungal biomass as previously described [35]. Fourteen days after inoculation the ITs of plants not used for the chitin assay were recorded for the lines in each of the four genotypic classes (“null”, *Sr12*, *Lr34*, and *Sr12* + *Lr34*).

### Genetic mapping and DNA markers

DNA was extracted from young leaves of RILs/DH lines in the RL, CS, and KN populations using a modified ammonium acetate extraction [36]. DNA was isolated from MN population using a rapid extraction protocol [12, 37]. RILs from RL population that were resistant or susceptible to *Pgt* in the field were used to construct DNA bulks to perform bulked segregant analysis (BSA) [38]. RL90, RL6058 and the resistant and susceptible bulks were tested with SNP markers using a 9K iSelect custom wheat SNP array [39]. SNP markers were analyzed using GenomeStudio software (Illumina Inc., San Diego, USA). Markers that were polymorphic between the parental lines and between the bulks were assessed for their association with stem rust severity in the entire RL population. QTL analysis was performed with QTL IciMapping software [40] using the markers identified by BSA to determine if they were associated with field stem rust resistance. SNP markers from the iSelect array associated with stem rust resistance were converted to KASP assays [41] for convenient mapping in populations (S1 Table).

DNA sequences associated with SNP markers were positioned on the 3B pseudomolecule [42]. The genomic sequence defined by SNP markers that flanked the *Sr* gene was searched for nucleotide-binding leucine repeat like-sequences (NB-LRR) as members of this class commonly encode disease resistance genes. SNP markers were developed from the NB-LRR-like sequences (S1 Table) and mapped in the RL-F_2_ recombinants along with the SNP markers from the 9K iSelect array. A marker from one NB-LRR-like sequence (*NB-LRR3*) that co-segregated with stem rust resistance was tested on the CS, KN, and MN populations.

The CS population was genotyped with SSR markers on chromosome 3B [43–46] and the KASP markers (above) converted from the 9K iSelect custom SNP array [39] that were mapped in the RL population. The *NB-LRR3* SNP marker was added to the CS genetic map and the association with stem rust resistance was determined.

A whole-genome map was constructed in the KN population using Diversity Array Technologies (DArT) markers [47], SSR markers [43–46] and SNP markers from the 9K iSelect custom SNP array [39] to map several qualitative and quantitative traits, including field resistance to Ug99 (McCartney, unpublished). In this study, the *NB-LRR3* marker was added to the KN genetic map to determine the association with stem rust resistance.

Linkage maps of the chromosome 3B region were constructed in the RL, CS and KN populations using MapDisto [48]. The map position of the maximum LOD for reduction in stem rust severity, percent variation explained (PVE), and additive effect was calculated for the above populations in each environment using QTL IciMapping [40]. The *NB-LRR3* was added to a previously published genetic map of *Sr12* in the MN population [28]. *NB-LRR3* was also tested on a panel of North American and Australian germplasm.

## Results

### Stem rust phenotyping of populations in the field

**Fig 1:**
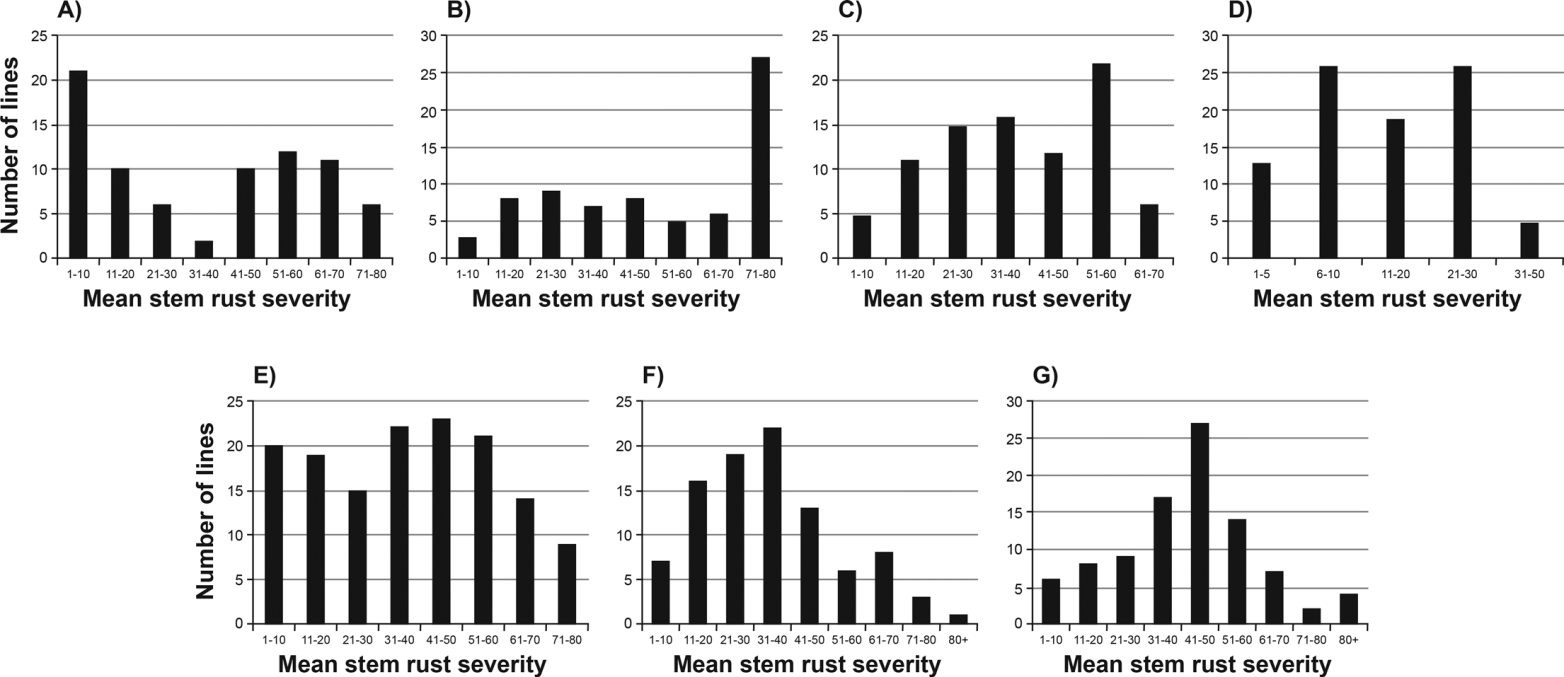
Distributions of stem rust severities in the A) RL population tested in Morden, Canada in 2014, B) RL population tested in Morden, Canada in 2015, C) RL population tested in St. Paul, USA in 2013, D) RL population tested in Njoro, Kenya in 2013, E) CS population tested in Morden, Canada in 2014, F) KN population tested in Njoro, Kenya in 2013, and G) KN population tested in Njoro, Kenya in 2014.

The RL population was evaluated for response to stem rust in Morden, Canada in 2014 and 2015, in Njoro, Kenya in 2013 and in St Paul, USA in 2013. The KN population was scored in Njoro, Kenya in 2013 and 2014 and the CS population in Morden, Canada in 2014. The field trials for the RL, CS, and KN populations were uniform in maturity and showed a full range (resistant to susceptible) of stem rust severities regardless of the location of the trial (Fig 1). Thus, adequate disease pressure resulted in phenotypic separation of lines within each population. With the exception of the RL population in Morden, field tests showed a continuous distribution for stem rust severity. The RL population resembled a bi-modal distribution in Morden in 2014. The rust severity across all plots in the RL90 test in Morden increased from 36.3 in 2014 to 52.5 in 2015 which was reflected in the different frequency distributions. However, the ranking of RL lines according to their response to stem rust was largely consistent across years resulting in heritability of 0.76. The overall heritability of response to stem rust for the RL population across four environments was 0.79 and 0.87 for the KN population across two environments. The CS population was tested in one environment and showed a heritability of 0.86 between replicates.

### DNA markers and genetic mapping of stem rust resistance in RL population

**Fig 2:**
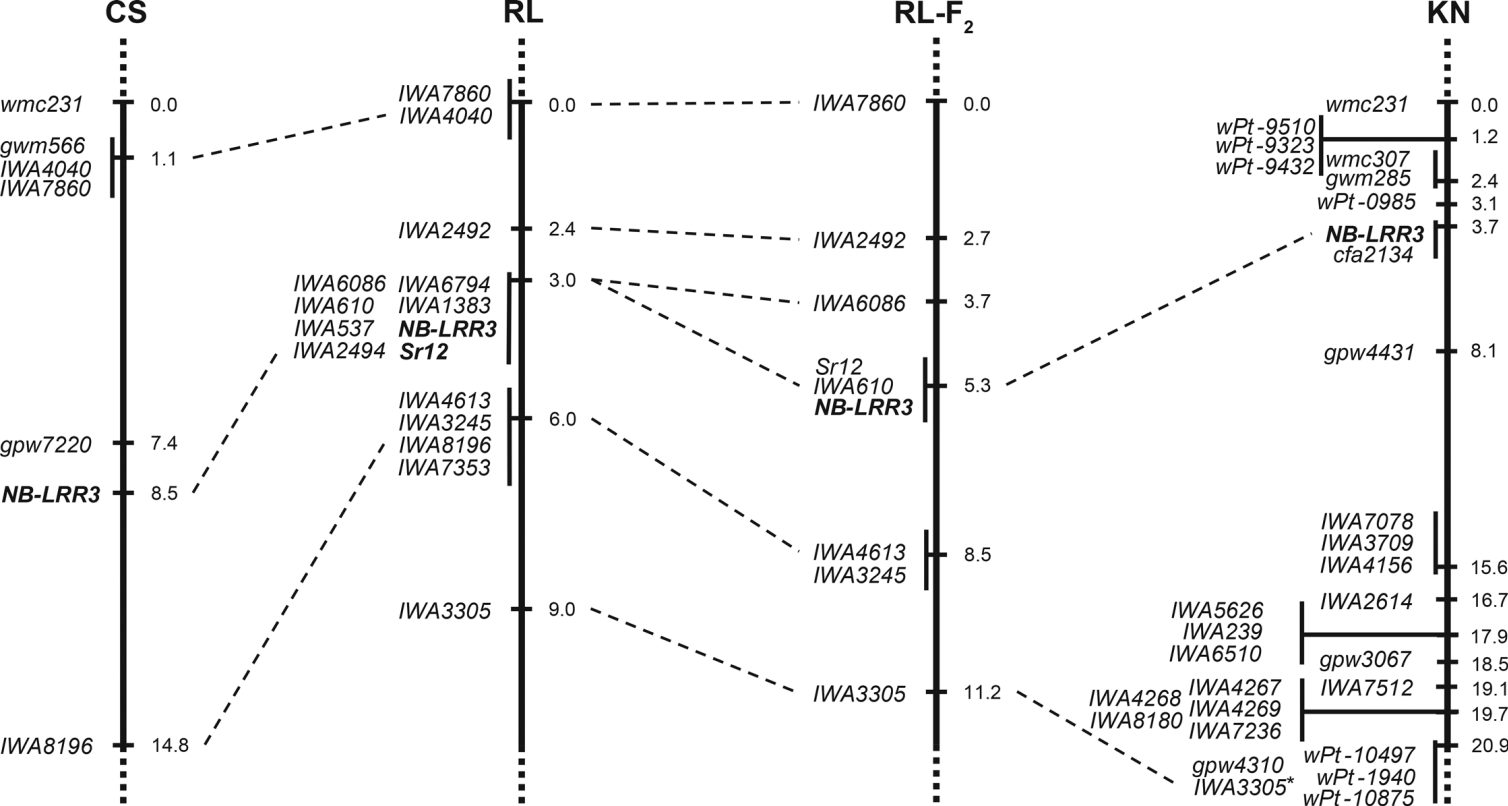
Genetic maps of the proximal region of chromosome 3BL in the Chinese Spring/RL6058 (CS) DH, RL90/RL6059 (RL) RIL, RL90/RL6058 F_2_-equivalent (RL-F_2_), and Kenyon/8615MN-2137 (KN) RIL populations. Map positions are in centi-Morgans. *There were 11 additional SNP markers that co-segregated with *IWA3305* in the KN population–*IWA3306*, *IWA4427*, *IWA4429*, *IWA4847*, *IWA5775*, *IWA5874*, *IWA6221*, *IWA6492*, *IWA6493*, *IWA7132*, and *IWA7388*.

In the RL population, field scores from USA and Kenya were used to select the most resistant and susceptible lines for BSA. SNP markers from the 9K iSelect array that differentiated the parents and the bulks were located at chromosomes 3B and 2B. These results suggested that these chromosomes carried *Sr* gene(s) responsible for the field resistance in RL6058 (S2 Table). Some of these SNP markers were converted to KASP assays and mapped on the RL population. While there was a weak association between markers on chromosome 2B and stem rust severity, the association was much stronger with the markers on chromosome 3B. A partial linkage map of the centromeric region of chromosome 3B was constructed and the marker/trait association was calculated for each marker. The association between genotype and stem rust severity was strongest with SNP marker *IWA610* in all four environments (PVE = 50.6% to 83.9%) (Table 1). A subset of the RL population was tested at the seedling stage using Australian *Pgt* race 98–1,2,(3),(5),6. The parental lines RL90 and RL6058 showed infection types (ITs) “3” and “12^-^”, respectively, and similar ITs were produced by the susceptible (“3” to “33^+^”) and resistant RILs (“12” to “2”). SNP markers *IWA6086*, *IWA610* and *IWA4613* that were previously associated with field resistance were also associated with seedling resistance. Markers *IWA6086* and *IWA3245* were predicted to flank the locus. Because the race-specific stem rust resistance gene *Sr12* was previously mapped to the centromeric region of chromosome 3B [12, 29], it is possible that seedling resistance detected by race 98–1,2,(3),(5),6 corresponded to *Sr12*. The hypothesis was supported by testing the RL population at the seedling stage with the Canadian *Pgt* race MCCFR which is avirulent on *Sr12*. The parental lines RL90 and RL6058 showed infection types (IT) of “33^+^” and “;” respectively, and the susceptible and resistant progeny lines showed ITs of “33^-^” to “33^+^” and “;” to “12^-^” respectively. The segregation of stem rust response (45 resistant: 49 susceptible) fitted the ratio for a single gene (χ^2^_1:1_ = 0.17, *p* = 0.68) and co-segregated with *IWA610* on chromosome 3B (Fig 2). The results indicate that the genetic interval carrying stem rust resistance which was observed under field conditions in USA, Kenya and Canada overlapped with the map position of *Sr12* as determined by seedling tests.

**Table 1:** Statistics for QTL analysis (interval mapping) of stem rust severity in the RL, CS, and KN populations.

Population	Environment	Position (cM)^a^	LOD^b^	PVE(%)^c^	Add^d^
RL	Canada 2014	3.0	28.98	83.93	-24.31
RL	Canada 2015	2.4	19.17	70.16	-21.11
RL	Kenya 2013	3.6	14.14	56.26	-9.12
RL	USA 2013	3.5	20.29	69.94	-14.40
CS	Canada 2014	7.0	12.09	33.68	-13.19
KN	Kenya 2013	3.1	4.13	18.16	-7.88
KN	Kenya 2014	3.1	5.16	22.12	-9.30

^a^ The “position” refers to the position on the genetic maps of chromosome arm 3BL for each population shown in Fig 2.

^b^ The significant LOD thresholds for the RL and CS populations were set at 3.0 as permutation analysis is inappropriate in the absence of a full genome map. However, if thresholds from a permutation test were used, the significance threshold would be less stringent. Permutation analysis (1000 permutations) was performed for the KN population and the significant LOD threshold was 3.4 for 2013 and 3.5 for 2014.

^c^ Percent variation explained for stem rust severity by the QTL on chromosome arm 3BL.

^d^ The additive effect that the resistant allele has on stem rust severity.

### Physical mapping of stem rust resistance

**Fig 3:**
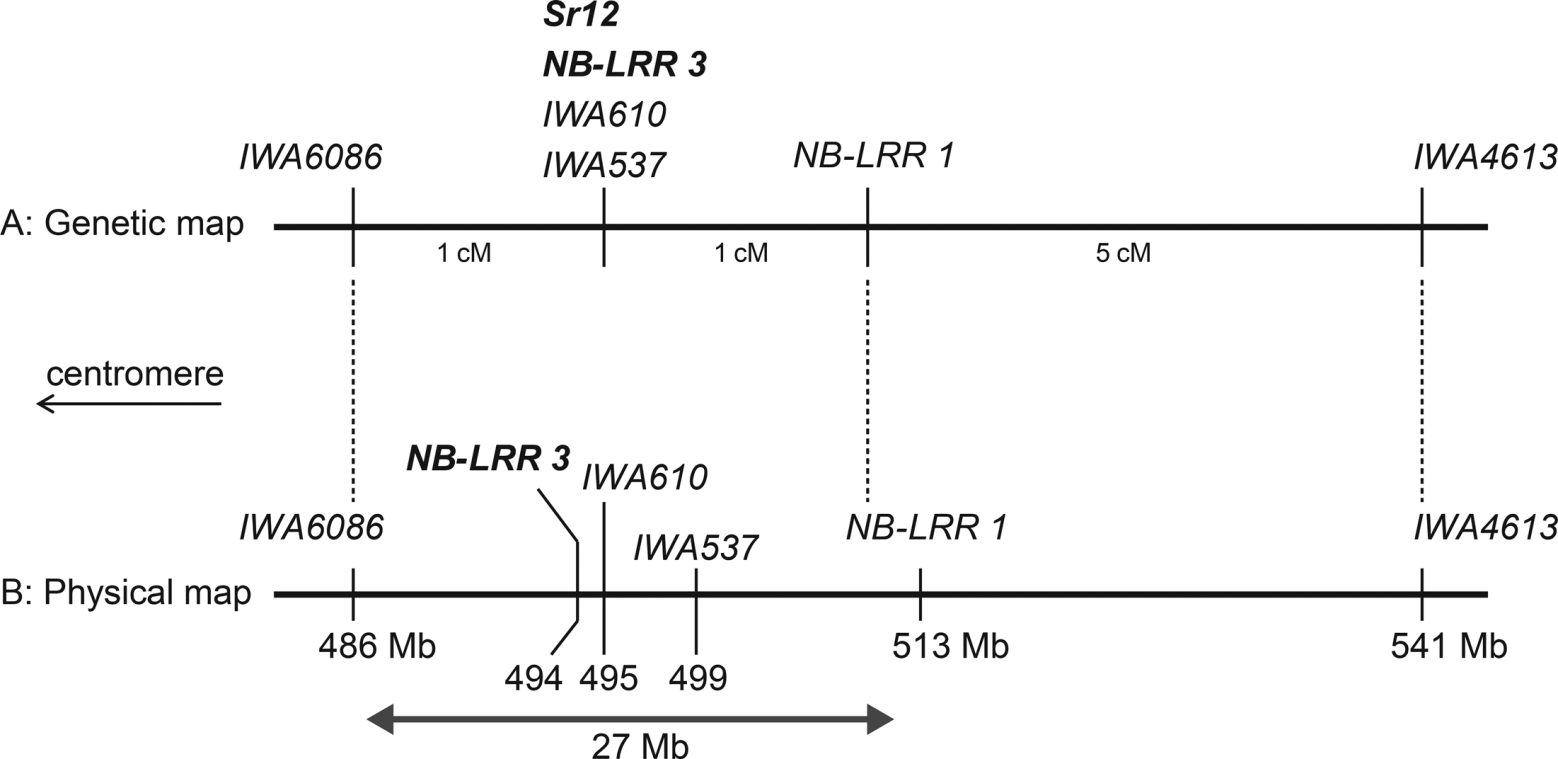
Alignment of the RL-F_2_ genetic map (A) with the physical map derived from the chromosome 3B pseudo-molecule (B).

SNP markers *IWA6086* and *IWA3245*/*IWA4613* defined the genetic interval carrying the resistance on chromosome 3B. The RL-F_2_ consisted of 110 F_2_-equivalent plants and was screened with flanking markers to identify individuals that were recombinant in the region. Fifteen recombinant F_2_ plants were identified which were progeny-tested in F_3_ with *Pgt* race 98–1,2,(3),(5),6 at the seedling stage (see above). Stem rust resistance was mapped within the genetic interval flanked by *IWA6086* and *IWA4613* and confirmed to co-segregate with *IWA610* (Fig 2). Flanking SNP markers were placed on the genome sequence of the chromosome 3B pseudo-molecule of ‘Chinese Spring’. The DNA sequence of approximately 55 Mb between *IWA6086* and *IWA4613* was searched for sequences with homology to members of the nucleotide binding-leucine rich repeat (NB-LRR) resistance gene family. Two NB-LRR sequences were identified at position 513 Mb and 494 Mb on chromosome 3B (Fig 3). The NB-LRR sequence at position 513 MB was separated from the seedling resistance by 2 recombinants, while a KASP marker developed from the partial NB-LRR sequence at position 494 Mb (*NB-LRR3*) co-segregated with seedling resistance. Two SNP markers, *IWA610* and *IWA537*, co-segregated with *NB-LRR3*; however, physical mapping determined their order on the chromosome 3B pseudo-molecule (Fig 3). The physical interval in ‘Chinese Spring’ that corresponded to the resistance gene interval in RL6058 was approximately 27 Mb and mapped to the centromeric region on the long arm of chromosome 3B as determined by the analysis of ditelosomic aneuploid lines of ‘Chinese Spring’. It is likely that the seedling resistance mapped here corresponds to *Sr12* in RL6058. ‘Chinese Spring’ contained a partial NB-LRR gene in this region which may be homologous to a candidate gene for *Sr12* in RL6058.

### NB-LRR3 associated with stem rust resistance in other populations

Markers from chromosome 3B were also tested for association with stem rust severity in the CS population. The *NB-LRR3* marker was added to the genetic map for the CS population in which it co-segregated with markers that showed the best association with field resistance (Table 1; Fig 2). The KN RIL population was tested in Kenya for resistance to Ug99-type stem rust in the field. A single QTL was identified on chromosome 3B. The *NB-LRR3* marker was added to the genetic map and identified the same chromosome region that was responsible for field stem rust resistance in the KN population as identified in the RL and CS populations (Table 1; Fig 2). The peak of the QTL corresponded to the map location of *NB-LRR3* which is consistent with the RL and CS populations.

Furthermore, *NB-LRR3* was added to a genetic map of chromosome 3B in the MN population that was previously used to map *Sr12* [12]. In this population *NB-LRR3* also co-located with a major QTL for stem rust resistance that was observed under field conditions on chromosome 3B and co-segregated with race SCCSC seedling resistance, which was presumably *Sr12*. Between the RL and MN populations there were a total 248 RILs tested which is equivalent to 496 opportunities for crossing-over, yet no recombinants were observed between *Sr12* and *NB-LRR3*.

### *Lr34* effect on stem rust resistance

**Fig 4:**
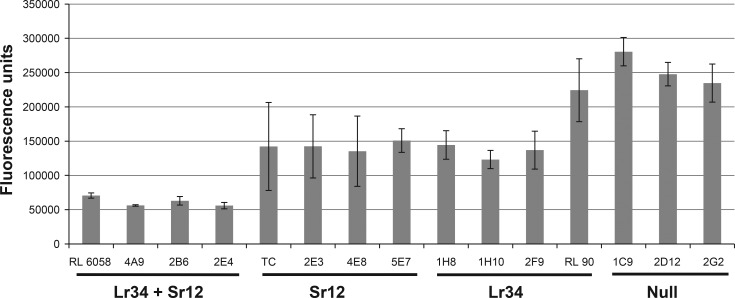
Amount of *Sr12*-avirulent *Pgt* race 98–1,2,(3),(5),6 infection at the seedling stage after 10 DPI in four genotypic classes of homozygous lines from the RL90/RL6077 population as determined by an assay measuring chitin abundance in the leaf tissue. TC represents ‘Thatcher’.

Previous reports showed that the combination of *Lr34* with additional genes from ‘Thatcher’ can contribute to enhanced stem rust resistance under field conditions [12, 26]. Here, we have quantified possible interactions by examining the effect of *Lr34* on *Sr12* at the seedling stage. Homozygous sister lines from the RL90/RL6077 population that represented four genotypic categories; null (neither *Lr34* nor *Sr12* were present), *Lr34*, *Sr12* and *Lr34*+*Sr12* were evaluated for stem rust infection using the Australian *Pgt* race 98–1,2,(3),(5),6. *Lr67*, present in RL6077, was absent in all of the lines evaluated. The level of stem rust infection was determined by measuring chitin levels in leaf tissue at 10 DPI [35]. Single gene lines carrying either *Lr34* or *Sr12* showed a reduction in stem rust infection compared to null lines but lines with both genes reduced infection levels even further (Fig 4) indicating that the effect of *Lr34* and *Sr12* on stem rust resistance in response to an avirulent race is additive. *Lr34* by itself reduced fungal biomass and resulted in lower IT scores compared to null lines. Macroscopic symptoms at 14 DPI reflected differences in fungal biomass, where “null” lines showed IT = “3”, lines with either *Sr12* of *Lr34* showed IT = “2”, and lines with both genes showed IT = “11^-^”. The same lines were also tested in North America at the seedling stage and under field conditions. Lines carrying both genes were also more resistant than single gene lines when tested in the field in Minnesota in 2014 and in Canada in 2015 (Table 2). However, using US races *Lr34* by itself did not contribute to stem rust resistance at the seedling stage or in the field, indicating that stem rust resistance involving *Lr34* and *Sr12* is epistatic. Lines with *Sr12* did exhibit significantly reduced stem rust severities in response to the bulk inoculum that included some *Pgt* races that were avirulent to *Sr12*.

**Table 2:** Seedling and field stem rust responses of lines from the RL90/RL6077 population grouped by genotypic class.

			Seedling IT	Mean stem rust severity			
Line^a^	*Lr34*	*Sr12*	SCCSC	US 2014	SE	Canada 2015	SE
RL90/RL6077 lines	-	-	3^+^	55.0	1.9	72.2	2.22
RL90/RL6077 lines	-	+	0;	25.0	3.3	65.6	2.22
RL90/RL6077 lines	+	-	2 to 3^+^	50.0	5.0	70.0	3.33
RL90/RL6077 lines	+	+	0	16.0	1.9	30.6	5.47
Thatcher	-	+	0	5.0		15.0	2.89
RL6058	+	+	0	1.0		5.0	1.15
RL6077	-	+	0	1.0		5.2	2.09
RL90	+	-	3	40.0		60.0	10.00

^a^ RL6077 carries *Lr67* which likely contributed to a low stem rust severity, however none of the RL90/RL6077 lines carried *Lr67*.

### Allele survey of linked SNP markers

SNP markers were identified that are tightly linked to stem rust resistance on chromosome 3B, including the marker *NB-LRR3*. To evaluate the usefulness of the *NB-LRR3* marker for marker-assisted selection, the allele frequency was determined in ‘Thatcher’ derivatives and historic North American germplasm with known *Sr12* status. In these lines, the marker genotype corresponded with the presence/absence of *Sr12* including for ‘Iumillo’ durum, the donor line for *Sr12* (Table 3). We also tested the allele frequency of *NB-LRR3* and other linked SNP markers in 92 Australian cultivars of unknown *Sr12* status. The ‘Thatcher’ allele of *NB-LRR3* and *IWA610* was present in approximately 50% of lines tested (S3 Table). The ‘Thatcher’ haplotype at five linked SNP marker loci was present in more than one third of cultivars, suggesting that this haplotype is relatively common in Australian germplasm. The frequency of *Sr12* in these lines is unknown.

**Table 3:** Allele survey of *NB-LRR3* marker and seedling IT with *Pgt* race 98-1-2,(3),(5),6 on selected ‘Thatcher’ derivatives and historic North American germplasm.

Line/Cultivar	Seedling IT	Sr12	*NB-LRR3* KASP
Thatcher	1+	+	+
Iumillo durum	1-	+	+
Marquis	2,2+	-	-
RL6077	; 1-	+	+
RL90	3	-	-
RL6071	2+	-	-
RL6058	;1	+	+
Canthatch	1-	+	+
Columbus	1+	+	+
Neepawa	1-	+	+
Napayo	1 C	+	+
Red Fife	3	-	-
NewThatch (*Sr2*)	1+	+	+
Glenlea	;	-	-
Prelude	;	-	-

The *NB-LRR3* marker was tested on 97 wheat cultivars and lines developed in or introduced into Canada. In total, 50 lines carried the ‘Thatcher’ allele of *NB-LRR3* while 47 did not (S3 Table). The influence of ‘Thatcher’ in the genealogy of Canadian Western Red Spring (CWRS) wheat is apparent in the results. Most of these cultivars that carry the Thatcher allele of *NB-LRR3* have Thatcher in their pedigrees (S3 Table) [8]. The cultivars from the Canadian Prairie Spring (CPS) class of wheat commonly carried the ‘Thatcher’ allele of *NB-LRR3* despite being more genetically distinct from the CWRS cultivars. The ‘Thatcher’ allele was also present in some of the soft white spring and hard red winter cultivars. As in the Australian lines above, the distribution of *Sr12* in this set of cultivars is largely unknown.

## Discussion

### Relationship of *Sr12* with field stem rust resistance

In this study, stem rust resistance in the field co-located with the race-specific *Sr12* gene on chromosome arm 3BL in the RL, RL-F_2_ and MN populations. Previously, it was uncertain which arm of chromosome carried *Sr12* [29], however this has been resolved by the genetic and physical maps presented here. The *NB-LRR3* marker, which explained a large proportion of phenotypic variation for stem rust resistance in the field, co-segregated with *Sr12* in these populations. However, it is unknown if *Sr12* is responsible, in whole or in part, for the APR observed on chromosome arm 3BL. *Sr12* is ineffective at the seedling stage to field races in Kenya [3] and is largely ineffective to North American races of *Pgt*. In North America the mixture of isolates used for inoculating field trials included some isolates that were avirulent to *Sr12*. In Canada one out of seven races (14%) was avirulent to *Sr12* and in the USA two of the six races (33%) were avirulent to *Sr12*. This could explain why the effect of the *Sr12* region was stronger in North America than in Kenya for the RL population (Table 1), however this does not explain the effect of the *Sr12* region on APR when races virulent to *Sr12* are present. There are two possible explanations: APR and *Sr12* seedling resistance are controlled by independent genes on 3BL, and/or under field conditions *Sr12* can provide APR against races that are virulent in the seedling stage. There are reports that describe APR to stem rust in ‘Thatcher’ including the association of the centromeric 3B region, thereby lending support to the independent gene hypothesis [49]. Alternatively, *Sr12* may contribute APR by itself or through interactions with other genes. Stem rust resistance in ‘Thatcher’ has been studied extensively but the resistance was found to be complex and difficult to dissect [10].

Recently, a major QTL on chromosome arm 3BL in ‘Thatcher’, which was associated with *Sr12*, required other unlinked genes to confer effective stem rust resistance in the field [12]. It is possible that gene interactions are relatively common but remain poorly understood. For example, the Canadian cultivar ‘Pasqua’ shows no visual symptoms to leaf rust (*Puccinia triticina* Eriks.) in the field in most seasons and has always been very resistant since its release [22, 50]. ‘Pasqua’ carries *Lr11*, *Lr13*, *Lr14b*, *Lr30*, and *Lr34* for leaf rust resistance (Dyck 1993). Analysis of these genes in near-isogenic lines in the greenhouse and in the field has shown that none of the genes confers resistance singly to a level near the resistance observed in ‘Pasqua’ (B. McCallum, personal communication). The genes *Lr11*, *Lr13*, *Lr14b*, and *Lr30* confer race-specific resistance while *Lr34* confers race-nonspecific resistance that is of a quantitative nature. However, reconstruction of the *Lr* gene combination found in ‘Pasqua’ and in various two, three, and four gene combinations has shown that genes that contribute little to resistance in isolation can still contribute to APR in the field when combined with other genes, particularly in combination with *Lr34*. For example, lines with *Lr11* singly showed an average leaf rust severity of 52% over four season and lines with *Lr34* singly showed an average severity of 26%, however lines with *Lr11* and *Lr34* combined had an average severity of 2% (B. McCallum, personal communication). Major genes are typically assessed for effectiveness by seedling tests; however, relatively little emphasis is placed on their utility in “field resistance”. It appears that some defeated or partially defeated rust resistance genes may play a role in APR when combined with other genes.

### *Lr34* effect on stem rust resistance

A goal of this study was to identify and genetically map stem rust resistance in ‘Thatcher’ that is expressed or enhanced in the presence of *Lr34*. In the RL and CS populations, which were fixed for *Lr34*, a region of chromosome arm 3BL was identified that contributed to resistance in the field. To test for interactions, sister lines were developed from a population that was segregating for *Lr34* and *Sr12* in the RL90/RL6077 population. Using these lines we showed that *Lr34* can reduce stem rust infection at the seedling stage, although *Lr34* is not known to confer stem rust resistance in seedling assays. Using a chitin assay to determine the fungal biomass before macroscopic symptoms became visible provided a more sensitive assay to study the early infection process and the effect of *Lr34*. Field tests of these lines showed that stem rust severity in each genotypic group agreed with both the seedling test for IT and fungal biomass (Fig 4; Table 2). The field resistance on chromosome 3BL did not depend on the presence of *Lr34*, as demonstrated by the results obtained from the KN population which lacked *Lr34* (Table 1).

Previously, one study identified resistance from ‘Thatcher’ located on chromosome arm 2BL that interacted with *Lr34* but did not report resistance on chromosome 3B [26]. In a follow-up study, further mapping of the resistance on chromosome arm 2BL found that the peak of the QTL for resistance was marked by the SSR *wmc175* [25]. This marker is closely associated with the *Sr9* locus [27, 28]. In another study analysing the stem rust resistance in ‘Thatcher’ four QTLs were identified, which were located on chromosomes 3B, 1A, 7DS, and 2BS but no QTL was detected on chromosome arm 2BL [12]. Of the QTL reported in that study, only the QTL on chromosome 3B was significant in all three field environments and it corresponded to the mapped location of *Sr12* [12]. The QTL on 7DS corresponded to *Lr34*, which interacted with the 3B resistance by reducing rust severity in the field. The association between the *Sr12* locus and APR in the field is consistent with our findings. Here, we also identified markers on 2B that were associated with resistance, but because of the relatively weak effect from 2B which was probably masked by the strong 3B effect, it was not possible to accurately map the 2B resistance and evaluate if this region interacted with *Lr34*.

In the RL population, association of the *NB-LRR3* marker (and other co-segregating markers) with stem rust resistance was high in all three environments, particularly in Canada. While one of the races (MCCFR) used in the *Pgt* field inoculum in Canada is avirulent to *Sr12*, approximately 85% of the field inoculum was composed of six races that are virulent to *Sr12*. Similar results were observed for the CS and KN populations as in the RL population, although the PVE values were lower (Table 1). If *Lr34* does enhance the resistance on 3BL as previously reported [12], it is possible that the effect of the 3BL resistance was lower in the KN population due to the absence of *Lr34* in the population. However, the additive effect of the *Sr12* region was similar for the KN population and the RL population in Kenya. Like the RL population, the CS population was fixed for *Lr34*; however, the *NB-LRR3* marker explained much less of the phenotypic variation in the CS population compared to the RL population. In both populations the resistant parent was RL6058 (a ‘Thatcher’ near-isogenic line carrying *Lr34*) thus the genetic basis of resistance should be the same. However, ‘Chinese Spring’, the susceptible parent in the CS population matures late compared to Thatcher-derived lines and as a result the DH lines varied in their maturity dates. It is most likely that the variation in maturity affected the stem rust severity scores in the CS population.

### Candidate gene for *Sr12*

Physical mapping localized a partial NB-LRR-like sequence within the interval in the ‘Chinese Spring’ reference sequence that corresponded to the region in RL6058 carrying *Sr12*. The NB-LRR (NB-LRR3) sequence in ‘Chinese Spring’ was predicted to encode only part of the 3’ half of a full length NB-LRR gene. Although the partial NB-LRR gene is expressed in seedling leaves of RL6058, we have not yet been able to isolate a full length transcript. It is uncertain if the partial NB-LRR sequence is closely related to a full length NB-LRR gene and candidate gene for *Sr12*. Given the large physical interval in ‘Chinese Spring’, it is possible that RL6058 carries additional NB-LRR genes in the corresponding interval which are absent in ‘Chinese Spring’. Our results reinforce the notion that sequence information from resistant donor germplasm is often required to identify candidate genes for disease resistance.

## Conclusion

The goal was to improve our understanding why Thatcher-*Lr34* (RL6058) is significantly more resistant to stem rust than ‘Thatcher’. Our study identified a proximal region on chromosome 3BL as the most significant factor for stem rust resistance in populations which were fixed for *Lr34*. Unexpectedly, this region was also responsible for the resistance to race Ug99 in Kenya identified in the KN population which lacked *Lr34*. Further analysis of the RL, RL-F_2_, and MN populations showed that *Sr12* is either responsible or closely linked to the field resistance observed in Kenya, the USA, and Canada. Seedling tests demonstrated that stem rust resistance on 3BL is enhanced in the presence of *Lr34*. Given these results, selection of *Sr12* region could provide a component of durable stem rust resistance in breeding programs and the *NB-LRR3* marker is a useful tool to assist in the selection process. Although the ‘Thatcher’ allele of this marker occurs in approximately 50% of Australian and Canadian cultivars, the diagnostic value cannot be assessed until the *Sr12* status of these lines is determined. In the meantime, work continues to identify the *Sr12* resistance gene through mutagenesis and the sequencing of NB-LRR gene sequences from mutants. The work is expected to generate a gene-based marker for *Sr12* and shed light on the relationship of *Sr12* and the associated APR.

## Supporting Information

S1 Fig Allele survey of *NB-LRR3* marker of 60 Australian wheat cultivars showing the clustering of allele 1 (orange, Thatcher) and allele 2 (blue, non-Thatcher) using the KASP assay. (TIF)

S1 Table Primer sequences for SNP based markers that were mapped to chromosome 3BL using KASP assays. (XLSX)

S2 Table Markers identified by bulked segregant analysis from the 9k iSelect SNP array as being associated with field resistance to stem rust in the RL population. (XLSX)

S3 Table Allele survey of SNP markers linked to *Sr12* in Australian germplasm and Canadian (North American) germplasm. (XLSX)

## Abbreviations

APR: adult plant resistance
BSA: bulked-segregant analysis
SNP: single nucleotide polymorphism
QTL: quantitative trait locus
NB-LRR: nucleotide binding-leucine rich repeat
