# Supplementary figures and images for: Major Gene for Field Stem Rust Resistance Co-Locates with Resistance Gene Sr12 in ‘Thatcher’ Wheat
Source: PLoS One. 2016 Jun 16;11(6):e0157029. doi: 10.1371/journal.pone.0157029 (PMC4911119; doi:10.1371/journal.pone.0157029)

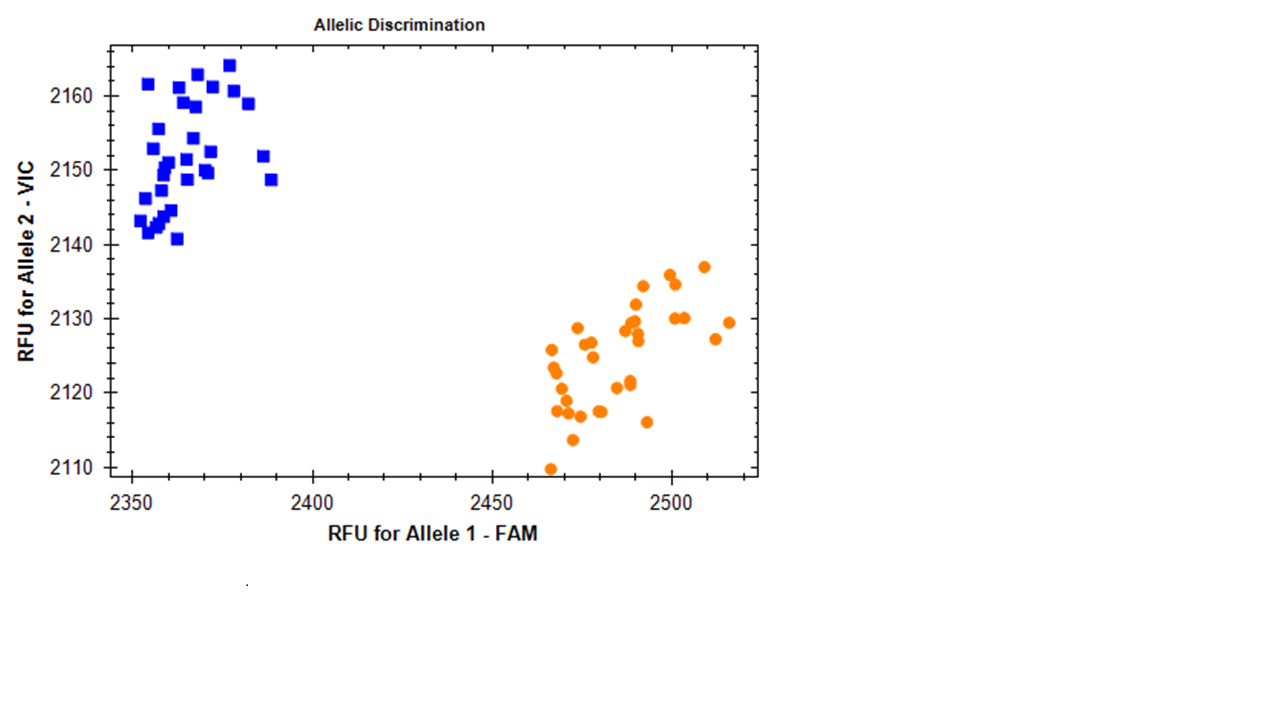

Supplement: S1 Fig — (TIF) [file pone.0157029.s001.tif]
